# Internalized Weight Bias and Disordered Eating: The Mediating Role of Body Image Avoidance and Drive for Thinness

**DOI:** 10.3389/fpsyg.2019.02999

**Published:** 2020-01-22

**Authors:** Rachel D. Marshall, Janet D. Latner, Akihiko Masuda

**Affiliations:** Department of Psychology, University of Hawai‘i at Mānoa, Honolulu, HI, United States

**Keywords:** internalized weight bias, disordered eating, eating disorders, body image, drive for thinness, internalized weight stigma, body image avoidance

## Abstract

Internalized weight bias has been linked with undesirable physical and psychological health outcomes, including disordered eating. Interventions have targeted internalized weight bias and associated outcomes, but little is known about underlying mechanisms of change. Existing treatment literature suggests that drive for thinness and body image avoidance may sustain the link between internalized weight bias and disordered eating. The present study aimed to determine if drive for thinness and body image avoidance mediated the relationship between internalized weight bias and disordered eating in an ethnically diverse sample. Participants included 225 female college students aged 18–49 years (mean age = 20.4 years, *SD* = 4.4), with a mean BMI of 23.3 kg/m^2^ who completed a computer-based survey for partial course credit. As expected, internalized weight bias was positively associated with disordered eating, and results supported the hypothesis of the mediating role of drive for thinness and body image avoidance. These results are important given the shortage of intervention efforts targeting internalized weight bias. Future intervention efforts aimed at reducing internalized weight bias and associated outcomes may benefit from simultaneously targeting drive for thinness and body image avoidance.

## Introduction

Internalized weight bias (IWB), the belief that negative stereotypes about weight apply to the self, is a significant concern in women, as it has been linked with a range of physical and psychological issues, including disordered eating (Durso et al., 2012, 2016). For example, IWB is associated with binge eating among treatment-seeking adults (Carels et al., 2010) and with dietary restraint among college women (Sienko et al., 2016). Over the course of treatment, IWB may moderate the efficacy of existing health interventions for women who are overweight, as participants with high IWB do not experience improvements in disordered eating (Mensinger et al., 2016).

Contemporary cognitive behavioral therapy (CBT) has attempted to offset the negative impact of IWB (Carels et al., 2014; Pearl et al., 2018). However, evidence regarding the mechanisms of change to offset the link between IWB and disordered eating remains sparse. Testing factors that are theorized to sustain the relationship between IWB and disordered eating is essential for understanding intervention efforts and improving their efficacy. Existing literature on CBT for eating disorders suggests that body image avoidance and drive for thinness may be underlying mechanisms of the iatrogenic link between IWB and disordered eating. The present cross-sectional study examined whether these two variables mediate the association between IWB and disordered eating.

### Drive for Thinness

According to CBT models, drive for thinness is a major risk factor for eating disorders. In treatment, drive for thinness is often a major target of intervention primarily because it is theorized to be an underlying variable that accounts for a range of eating disorder symptoms (Fairburn et al., 2003). Drive for thinness is associated with disordered eating; for example, individuals with eating pathology (e.g., anorexia nervosa) exhibit higher levels of drive for thinness than healthy controls (Garner et al., 1983). In addition, prior research has demonstrated a significant, positive association between IWB and drive for thinness (*r* = 0.47 and *r* = 0.56) among community samples of adults across the entire BMI spectrum (Durso and Latner, 2008; Pearl and Puhl, 2014). Given these associations, first, between drive for thinness and disordered eating, and second, between IWB and drive for thinness, it can be speculated that drive for thinness might serve as a mediator of the connection between IWB and disordered eating.

### Body Image Avoidance

Body image avoidance is another candidate that might explain the link between IWB and disordered eating. Body image avoidance is often defined as refraining from entering situations that elicit worry about physical appearance (Rosen et al., 1991). Examples of body image avoidance include avoiding reflective surfaces (e.g., mirrors), being weighed, wearing form-fitting clothes, and being photographed. Body image avoidance is an important behavioral manifestation of body image disturbance, which includes cognitive, affective, and behavioral expressions of body dissatisfaction (Stormer and Thompson, 1996; Pellizzer et al., 2017). Multiple studies have observed a positive association between body image avoidance, disordered eating, and negative attitudes about body weight and shape (Durso and Latner, 2008; Walker et al., 2018; Purton et al., 2019). Additionally, higher IWB is associated broadly with body image disturbances (Durso and Latner, 2008; Carels et al., 2010; Durso et al., 2016). Considering the existing associations between body image avoidance with disordered eating and body image disturbance with IWB, there is reason to predict that a behavioral expression of body image disturbance (i.e., body image avoidance) mediates the relationship between IWB and disordered eating.

#### Drive for Thinness and Body Image Avoidance as Two Underlying Mechanisms

Cognitive behavioral therapy literature emphasizes drive for thinness and body image avoidance as variables associated with both IWB and disordered eating. IWB significantly predicted drive for thinness among an overweight community sample (Durso and Latner, 2008). Additionally, a transdiagnostic model of eating disorders emphasized drive for thinness as a risk factor and maintaining factor for eating disturbances (Fairburn et al., 2003). Body image avoidance is an important behavioral manifestation of body image disturbance, which prior research indicates is positively associated with IWB (Durso and Latner, 2008; Carels et al., 2010). Amin et al. (2014) observed greater body image avoidance among a clinical sample with eating disorders than a non-clinical control group. Additionally, reduction in body image avoidance is a significant predictor of improvement in disordered eating (Pellizzer et al., 2019). Considering aforementioned findings, both drive for thinness and body image avoidance should be simultaneously explored as potential mediators of the relationship between IWB and disordered eating. Drive for thinness and body image avoidance are associated cognitive and behavioral factors, both of which have been independently linked with IWB and disordered eating behaviors. For example, a woman who reports high levels of IWB is likely to experience a strong drive for thinness, along with behavioral efforts to avoid or down-regulate her negative body image. As potential underlying mechanisms in the context of IWB, both the urge to become thinner and efforts to down-regulate negative body image may trigger and perpetuate symptoms of disordered eating.

### Present Study

Existing literature has demonstrated associations between IWB, disordered eating, drive for thinness, and body image avoidance. CBT models suggest that drive for thinness and body image avoidance are underlying mechanisms of eating disorder pathology that are activated in the context of IWB, which in turn results in greater disordered eating. Although researchers have examined associations among variables of present interest partially, the proposed multiple mediation model has not been fully investigated. The present cross-sectional study was designed to directly investigate the proposed multiple mediation model.

## Method

### Participants

Female students were recruited from a public university in Hawaii through an online research recruitment pool managed by the Department of Psychology. To be included in this study, participants had to identify as female, be 18 or older, and speak English.

### Procedure and Measures

The present research was approved by and was carried out in accordance with the recommendations of the University of Hawaii Institutional Review Board. All subjects gave written informed consent prior to participation in this study. Participants who enrolled in the study were asked to complete an anonymous online survey, including survey measures and demographic information. The following measures were used to assess internalized weight bias, disordered eating, drive for thinness, and body image avoidance.

#### Internalized Weight Bias

The Weight Bias Internalization Scale – Modified (WBIS-M; Pearl and Puhl, 2014) is an 11-item self-report measure of IWB. This measure is a revised version of the original Weight Bias Internalization Scale (WBIS) by Durso and Latner (2008). Responses were rated on a 7-point Likert scale ranging from “Strongly Disagree” to “Strongly Agree”. The IWBS-M demonstrated high internal consistency (α = 0.90) and was positively correlated with BMI in a validation study (Pearl and Puhl, 2014). Similarly, the WBIS-M demonstrated high internal consistency (α = 0.95) and was significantly correlated with BMI (*r* = 0.47, *p* < 0.001) in the present study.

#### Disordered Eating

The Eating Attitudes Test (EAT-26; Garner et al., 1982) is a 26-item self-report measure used to detect symptoms and concerns related to disordered eating. Responses were rated on a 6-point Likert scale ranging from “Always” to “Never”. The EAT-26 demonstrated high reliability in a study of its psychometric properties among females with (α = 0.90) and without (α = 0.83) anorexia nervosa (AN; Garner et al., 1982). Additionally, the EAT-26 was highly correlated with the original 40-item scale, EAT-40 (Garner et al., 1982). In the present study, the EAT-26 had high internal consistency (α = 0.88).

#### Drive for Thinness

The Eating Disorders Inventory - Drive for Thinness Subscale (EDI- Drive for Thinness; Garner et al., 1983) is a 7-item self-report scale that measures fear of weight gain and desire to lose weight. Responses were rated on a 6-point Likert scale ranging from “Always” to “Never”. The scale demonstrated high internal consistency among those who met criteria for AN (α = 0.85) and a female control sample (α = 0.85; Garner et al., 1983). Internal consistency of the EDI-Drive for thinness Subscale was high in the present sample (α = 0.92).

#### Body Image Avoidance

The Body Image Avoidance Questionnaire (BIAQ; Rosen et al., 1991) is 19-item self-report questionnaire created to measure avoidance of situations that elicit concern about physical appearance. Responses were rated on a 6-point Likert scale ranging from “Never” to “Always”. In a validation study of the BIAQ among female university students, the scale demonstrated good internal consistency (α = 0.89; Rosen et al., 1991). The questionnaire also had good internal consistency (α = 0.84) in the present sample.

### Data Analysis

Descriptive statistics were run on demographic information to summarize the age, ethnicity, relationship status, and weight history of participants in our sample. We calculated the mean and standard deviation for all scales, and then examined the nature of the associations among the variables using bivariate correlations.

In order to examine the indirect effect of internalized weight bias on disordered eating through drive for thinness and body image avoidance, separately, a mediation analysis was conducted using a bootstrapping procedure based on the recommendation of A. F. Hayes (2013) and MacKinnon et al. (2007). The parallel mediation analysis was computed using the PROCESS macro for SPSS, version 3.4 (A. F. Hayes, 2013). The bootstrapping procedure constructed bias-corrected confidence intervals based on 5,000 random samples with replacement from the full sample (see: Preacher and Hayes, 2004, 2008). Direct effects, indirect effects, standard errors, and confidence intervals were estimated based on the distribution obtained using the bootstrapping procedure. A significant indirect effect indicates that the effect of IWB on the outcome variable disordered eating is accounted for by a mediating variable (e.g., drive for thinness), while controlling for the other mediator (e.g., body image avoidance).

## Results

Participants (*N* = 294) were undergraduate women enrolled in introductory psychology classes who completed the present web-based survey. Of those, 36 participants were excluded from the study because they did not identify as female. An additional 20 participants were excluded from the study because they were under 18-years-old. In order to determine how to handle missing data, Little’s Missing Completely at Random test (Little, 1988) was run *X*^2^ (441, *N* = 225) = 447.28, *p* = 0.408. This non-significant finding indicates that data were missing completely at random and the pattern of missing values does not depend on data values. Thus, listwise deletion of missing data from 13 participants who were missing partial data was deemed appropriate and unlikely to bias study results. The final sample consisted of 225 participants ranging in age from 18 to 49 years old (*M* = 20.39, *SD* = 4.41) and ranging in BMI from 15.58 to 40.72 (*M* = 23.29, *SD* = 4.92). The sample identified as follows: 45.8% Asian (*n* = 103), 31.6% Caucasian (*n* = 71), 8% Native Hawaiian/Pacific Islander (*n* = 18), 2.7% Multiracial (*n* = 6), 2.2% Hispanic/Latina (*n* = 5), 1.8% African American (*n* = 4), and 6.2% other ethnicity (*n* = 14). The sample was reflective of the ethnic diversity of the city in which the research was conducted.

### Associations Among Study Variables

Pearson correlations were calculated among the study variables (see Table 1). IWB was positively correlated with disordered eating, drive for thinness, body image avoidance, and BMI. Additionally, disordered eating was positively correlated with drive for thinness, body image avoidance, and BMI. Drive for thinness was positively associated with body image avoidance and BMI. Last, body image avoidance was positively correlated with BMI.

**TABLE 1:** Pearson correlations between study variables.

**Variable**	**1**	**2**	**3**	**4**	**5**
1. Internalized weight bias (WBIS-M)	–				
2. Disordered eating (EAT-26)	0.54^∗∗^	–			
3. Drive for thinness (EDI subscale)	0.67^∗∗^	0.69^∗∗^	–		
4. Body image avoidance (BIAQ)	0.63^∗∗^	0.58^∗∗^	0.58^∗∗^	–	
5. BMI	0.47^∗∗^	0.22^∗^	0.32^∗∗^	0.23^∗∗^	–
Mean	37.65	10.09	5.15	27.54	23.29
SD	15.77	10.10	6.02	11.10	4.92

*^∗^*p* < 0.05, ^∗∗^*p* < 0.01; WBIS-M, weight bias internalization scale – modified; EAT-26, eating attitudes test; EDI, eating disorder inventory – drive for thinness subscale; BIAQ, body image avoidance questionnaire; BMI, body mass index; SD, standard deviation.*

### Mediation Analysis

**FIGURE 1:**
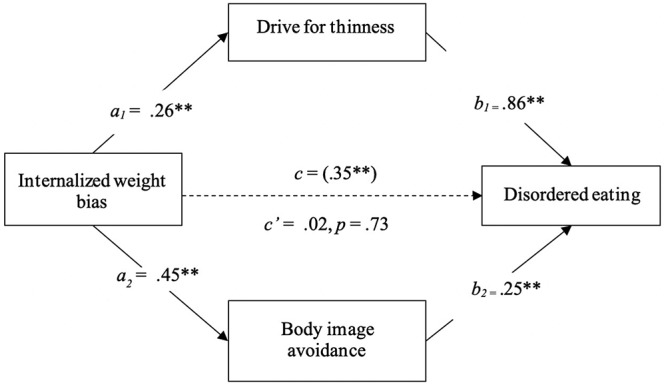
Mediation of the relationship between internalized weight bias and disordered eating by drive for thinness and body image avoidance. *N* = 225. The a-, b-, and c- paths indicate regression coefficients. c = total effect c’ = direct effect (when a and b are accounted for). Coefficients marked with an asterisk indicate significance, ^∗∗^*p* < 0.001, in terms of 95% bias-corrected confidence intervals that do not contain zero using 5,000 bootstrapped samples. Both mediator variables were considered simultaneously while controlling for the effect of one another.

A parallel mediation analysis was conducted using a bootstrapping resampling procedure to examine the indirect effect of IWB on disordered eating through drive for thinness and body image avoidance (see Figure 1). The size of the indirect effect of IWB on disordered eating through drive for thinness was 0.22 (*SE* = 0.04), and it was significant with a 95% confidence interval which did not include zero, 95% CI [0.15, 0.30]. This result indicates that the effect of IWB on disordered eating is mediated by drive for thinness. The size of the indirect effect of IWB on disordered eating through body image avoidance was 0.11 (*SE* = 0.03), and it was significant with a 95% confidence interval which did not include zero, 95% CI [0.05, 0.17]. This result indicates that the effect of IWB on disordered eating is also mediated by body image avoidance. The mediators, body image avoidance and drive for thinness, demonstrated significant a-paths and b-paths. The total effect of IWB on disordered eating (c-path) was reduced to non-significance (c’-path) when controlling for the effects of the mediators. Results indicate full parallel mediation of the relationship between IWB and disordered eating by drive for thinness and body image avoidance. In other words, the association between IWB and disordered eating is fully accounted for by drive for thinness and body image avoidance.

## Discussion

Internalized weight bias is cause for concern due to its association with numerous undesirable psychological and physical health issues, such as low self-esteem and disordered eating. Disordered eating is one health outcome of present interest, given the prevalence and persistence of eating disturbances among college women (Eisenberg et al., 2011). Little is known about mechanisms of change in interventions that attempt to reduce IWB and associated disordered eating. Guided by CBT models of eating disorders and eating disorder treatment, the present study was designed to determine whether drive for thinness and body image avoidance mediated the relationship between IWB and disordered eating. IWB, disordered eating, drive for thinness, body image avoidance, and BMI were positively associated with each other. Results support previous findings indicating positive association between IWB and disordered eating (e.g., binge eating) among samples of community and college adults (Puhl et al., 2007; Durso et al., 2016; Mehak et al., 2018). Findings also suggest the potential generalizability of such associations to subgroups of ethnically diverse women who are often not included in studies of body image and eating concerns.

A multiple mediation analysis showed that body image avoidance and drive for thinness together mediated the relationship between IWB and disordered eating, providing support for our hypotheses. These findings demonstrated that the relationship between IWB and disordered eating was not direct. Rather, the association between IWB and disordered eating was fully accounted for by other variables in this sample, namely, drive for thinness and body image avoidance. This finding is novel and important given the shortage of studies designed to reduce IWB and associated negative outcomes. Knowing the factors that maintain the relationship between IWB and disordered eating elucidates ideas for identifying or creating a potent intervention that addresses mediating factors in addition to IWB.

The current research lends itself to suggestions for future research. The present study included an ethnically diverse sample, including Asian, Caucasian, Native Hawaiian/Pacific Islander, Hispanic/Latina, and multiracial participants, but multiple groups were too small to permit conducting analyses by individual ethnic group. Examining the present mediation model across ethnic groups would allow researchers who work with diverse populations to confidently conceptualize relationships between variables of interest among those individuals. Future research would do well to recruit larger numbers of ethnically diverse individuals in order to facilitate greater generalizability of findings.

Findings from the current study have clinical implications for future prevention and intervention efforts targeting IWB and its association with eating disorder pathology. One might consider how the four variables of interest are experienced by a patient. A woman who attributes negative stereotypes (e.g., lazy, undisciplined) to herself due to her size or shape will likely avoid stimuli that trigger her body dissatisfaction while simultaneously making efforts to pursue a thinner body. In order to lose weight, the woman may avoid eating calorically dense foods with low nutritional value prior to experiencing unwanted binge eating episodes. This entire experience will reinforce the woman’s belief that she is undisciplined. Since drive for thinness and body image avoidance fully explain the association between IWB and disordered eating, interventions designed to prevent and/or reduce disordered eating among patients may be strengthened by explicitly targeting the mediators in addition to IWB. Such a multifaceted treatment would be closely aligned with cognitive-behavioral and acceptance-based intervention models, such as CBT, CBT-Enhanced (CBT-E; Byrne et al., 2011) and acceptance and commitment therapy (ACT; S. C. Hayes et al., 2006). CBT and ACT target IWB and disordered eating by undermining the negative impact of body image avoidance and drive for thinness. In light of current findings, use of interventions that challenge negative weight-based stereotypes (Carels et al., 2014), encourage body acceptance (Pearl et al., 2018), and facilitate cognitive defusion (i.e., identifying thoughts as products of the mind and not necessarily the truth; Lillis et al., 2009; Palmeira et al., 2017) may be beneficial for reducing IWB and associated negative outcomes.

Interventions for IWB often focus on individuals struggling with overweight or obesity (Berman et al., 2016; Levin et al., 2018; Pearl et al., 2018). Results from the present study show that while greater IWB is associated higher BMI scores, IWB also exists among females who are not overweight. This result is in line with other recent findings (Essayli et al., 2017), and indicates that females of varying sizes struggle with self-judgment based on their weight and shape. Women across the weight spectrum may be suffering from the deleterious psychological and physical impacts of IWB, including disordered eating. Future clinical interventions would do well to address IWB among individuals across the body weight spectrum.

Our findings should be interpreted in light of several notable limitations. First, the research explored a non-clinical sample. It is unknown if variables in the present study function similarly among those with clinical levels of eating disturbances. Additionally, an all-female sample was used because the variables of interest are thought to function differently between women and men (Smith et al., 2017; Puhl et al., 2018; Purton et al., 2019). The generalizability of the present findings to men is unknown. Future research that examines the relationship between IWB and disordered eating in men is encouraged. Additionally, the present study included a wider range of ages than is found in many college samples; however, the generalizability of the present findings to females under 18 or over 50 remains unclear. Another limitation includes the use of self-report measures. Participants may have been impacted by the social desirability bias, underreporting symptoms in order to present themselves favorably. The present study was correlational in nature, and for this reason, causal inferences cannot be made. Longitudinal and experimental research is needed to establish causation and determine the long-term effects of change in a particular variable (e.g., IWB).

The present study contributes to the study of IWB, body image, and disordered eating in meaningful ways. It confirms previously established associations among key variables in an ethnically diverse sample. Additionally, the current research contributes to the understanding of the relationship between IWB and disordered eating by indicating that drive for thinness and body image avoidance fully account for their association. CBT and ACT have independently demonstrated efficacy in reducing body image avoidance and drive for thinness, and both treatment modalities have also demonstrated preliminary success in reducing IWB. Future intervention efforts might do well to utilize CBT or ACT to target IWB, body image avoidance, and drive for thinness to prevent and/or reduce disordered eating.

## Data Availability Statement

The raw data supporting the conclusions of this article will be made available by the authors, without undue reservation, to any qualified researcher.

## Ethics Statement

The studies involving human participants were reviewed and approved by the University of Hawai‘i at Mānoa Institutional Review Board. The participants provided their written informed consent to participate in this study.

## Author Contributions

RM co-designed and conducted the study, analyzed the data, and wrote the manuscript. JL co-designed the study and edited the manuscript. AM collaborated on writing and editing the manuscript.

## Conflict of Interest

The authors declare that the research was conducted in the absence of any commercial or financial relationships that could be construed as a potential conflict of interest.
